# Epidemiology, clinical presentation and respiratory sequelae of adenovirus pneumonia in children in Kuala Lumpur, Malaysia

**DOI:** 10.1371/journal.pone.0205795

**Published:** 2018-10-15

**Authors:** Li Min Lim, Yen Yen Woo, Jessie Anne de Bruyne, Anna Marie Nathan, Sze Ying Kee, Yoke Fun Chan, Chun Wei Chiam, Kah Peng Eg, Surendran Thavagnanam, I-Ching Sam

**Affiliations:** 1 Department of Paediatrics, University Malaya, Kuala Lumpur, Malaysia; 2 Department of Medical Microbiology, University Malaya, Kuala Lumpur, Malaysia; 3 Department of Paediatrics, University Putra Malaysia, Serdang, Selangor Darul Ehsan, Malaysia; Lovelace Respiratory Research Institute, UNITED STATES

## Abstract

**Objectives:**

To describe the severity, human adenovirus (HAdV) type and respiratory morbidity following adenovirus pneumonia in children.

**Methodology:**

Retrospective review of children under 12 years of age, admitted with HAdV pneumonia, between January 2011 and July 2013, in a single centre in Malaysia. HAdV isolated from nasopharyngeal secretions were typed by sequencing hypervariable regions 1–6 of the hexon gene. Patients were reviewed for respiratory complications.

**Results:**

HAdV was detected in 131 children of whom 92 fulfilled inclusion criteria. Median (range) age was 1.1 (0.1–8.0) years with 80% under 2 years. Twenty percent had severe disease with a case-fatality rate of 5.4%. Duration of admission (p = 0.02) was independently associated with severe illness. Twenty-two percent developed respiratory complications, the commonest being bronchiolitis obliterans (15.2%) and recurrent wheeze (5.4%). The predominant type shifted from HAdV1 and HAdV3 in 2011 to HAdV7 in 2013. The commonest types identified were types 7 (54.4%), 1(17.7%) and 3 (12.6%). Four out of the five patients who died were positive for HAdV7. Infection with type 7 (OR 8.90, 95% CI 1.32, 59.89), family history of asthma (OR 14.80, 95% CI 2.12–103.21) and need for invasive or non-invasive ventilation (OR 151.84, 95% CI 9.93–2.32E) were independent predictors of respiratory complications.

**Conclusions:**

One in five children admitted with HAdV pneumonia had severe disease and 22% developed respiratory complications. Type 7 was commonly isolated in children with severe disease. Family history of asthma need for invasive or non-invasive ventilation and HAdV 7 were independent predictors of respiratory complications.

## Introduction

Human adenoviruses (HAdV) are a common cause of disease including respiratory infections, gastroenteritis, and conjunctivitis, particularly in children below 5 years of age[1] and HAdV accounts for about 5–10% of acute lower respiratory tract infections (ALRTIs). [2] Over 85 genotypes of HAdVs have been recognized by the Human Addenovirus Working Group based on bioinformatics analysis of complete genomic sequences.[3,4]. HAdV respiratory infections are predominantly caused by species B (including types 3, 7, 11, 14, 21 and 55), C (types 1, 2, 5, and 6) and E (type 4).

Recent worldwide epidemics of respiratory infections due to HAdV have resulted in renewed interest in this virus. The increase in cases of HAdV infection has also been reported in Asia. These include outbreaks due to established types such as HAdV type 7 (HAdV7), which have been reported in the community and in military and police camps between 2011 and 2013, in Taiwan, Singapore, China and Malaysia. [5–9] There have also been more recently emerging types such as HAdV55, described in China.[10]

Respiratory infections due to HAdV cause significant morbidity and mortality, with case fatality rates as high as 12%.[11] There is also a risk of up to 30% of developing long-term respiratory complications such as post-infectious bronchiolitis obliterans (PIBO) and bronchiectasis. [1,12]

The clinical and molecular epidemiology of HAdV respiratory infections and risk of complications are relatively understudied in developing countries. In this study, the aims were to a) describe the clinical presentation, severity, HAdV type and respiratory morbidity, b) determine risk factors associated with severe illness and the development of respiratory complications, c) describe the treatment modalities and outcomes, and d) assess quality of life of children admitted to our centre with acute HAdV pneumonia.

## Materials and methods

### Hospital setting

University Malaya Medical Centre (UMMC) is a government-funded teaching hospital in Kuala Lumpur, Malaysia. The paediatric building has 4 Paediatric wards, a Paediatric Intensive Care Unit (PICU) and a Neonatal Intensive Care unit. It also has its own Paediatric Emergency Department.

### Study design and ethical approval

This is a single centre, retrospective study conducted at UMMC. Ethical approval for this study was obtained from the institutional ethics committee (MECID.NO: 201410–659). Verbal consent was taken from patients' parents not on follow-up and information was obtained over the phone. Questionnaires were administered to parents whose children were on follow-up. The ethics committee approved this study.

### Study population

This study included all children younger than 12 years of age, admitted to UMMC with laboratory-confirmed HAdV pneumonia between 1^st^ January 2011 and 31^st^ July 2013. Children with pre-existing chronic lung disease, upper respiratory tract infections, those who could not be contacted or whose medical records could not be found were excluded. Eligible patients were identified as those whose respiratory samples tested positive for HAdV by the Department of Medical Microbiology of UMMC.

### Study definitions

Pneumonia was diagnosed based on history (either fever, cough and/or rapid breathing) and clinical evidence of pneumonia (either tachypnoea, chest recessions and/ or adventitious sounds upon lung auscultation) with radiographic signs (infiltrates or consolidation). HAdV infection was laboratory-confirmed by positive immunofluorescence assay and/or viral culture

Previous lung infection was defined as any child with a previous history of a lower respiratory tract infection (LRTI), which was verified by the presence of shortness of breath during that illness.

Severe HAdV pneumonia was defined as those requiring either invasive or non-invasive respiratory support, PICU care or illness resulting in death. Disseminated disease was defined as signs and laboratory evidence of involvement of 2 or more organ systems.

PIBO was diagnosed in children in the presence of either one of the following signs: tachypnoea, chest retractions, chest hyperinflation, wheeze, crepitations and hypoxaemia, for at least 30 days after the initial lung injury.[13] The diagnosis was made following high-resolution chest tomography showing some or all the following features: mosaic perfusion, vascular attenuation, atelectasis, expiratory air trapping, peri-bronchial thickening and bronchiectasis.[14] Respiratory complications include any form of chronic lung disease (including asthma) and death.

### Data collection

Medical records were obtained from the Medical Records Department and reviewed. Medical records and administration of the questionnaire was between 1^st^ February 2015 till 30^th^ September 2015. Data acquired included: socio-demographic data, anthropometric measurements, duration of admission, birth history, personal and family history of asthma and atopy, vaccination (which includes prior exposure to pneumococcal and/or influenza vaccines), treatment received (antibiotics and steroids) and clinical, laboratory and radiological investigations.

### Specimen collection, virus identification & typing

Nasopharyngeal aspirates (NPAs) are collected routinely in all children with LRTIs. Tracheal aspirates were only obtained from intubated children. All respiratory specimens were tested by direct immunofluorescence (IF) for 8 respiratory viruses: HAdV, influenza A and B, respiratory syncytial virus (RSV), metapneumovirus and parainfluenza viruses 1, 2 and 3 (D^3^ Ultra 8 DFA Respiratory Virus Screening & Identification Kit (Diagnostic Hybrids, USA). Viral isolation was performed by inoculating the NPAs into Madin Darby canine kidney (MDCK; ATCC number CCL-34), Vero (ATCC number CCL-81), A549 (ATCC number CCL-185), and HEp-2 (ATCC number CCL-23) cells. Cultures were incubated at 37°C with 5% CO_2_. Infected cells showing cytopathic effect within 10 days were harvested for IF.

HAdV isolates underwent further molecular typing. Viral DNA was extracted from adenovirus cultures with the QIAamp DNA Blood Mini Kit (Qiagen, Germany). The hypervariable regions 1–6 of the hexon gene were amplified and sequenced as previously described[15] with M13 universal priming tails added to the primers to facilitate sequencing.[16] Sequencing was carried out by First BASE Laboratories (Selangor, Malaysia). Sequences were edited using Geneious R7 (Biomatters, New Zealand) and aligned with relevant sequences from GenBank. MEGA7 [17] was used to construct phylogenetic trees by maximum likelihood with 1000 bootstrap reiterations, using the general time reversible model with gamma distribution of evolutionary rates and invariant sites. Separate trees were constructed for sequences of HAdV-B and HAdV-C. The sequences reported in this study were deposited into GenBank with accession numbers KU145006-KU145113.

### Treatment modalities

All children received standard treatment for pneumonia, which included supportive care, and where deemed necessary, invasive or non-invasive ventilation, inotropes and antibiotics. Intravenous immunoglobulin (IVIG) was given to children with persistent fever and intravenous pulse methylprednisolone (MTP) was given to children with significant disease i.e. rapidly increasing respiratory distress, at the discretion of the treating physician.

### Respiratory sequelae

Parents of discharged children with HAdV pneumonia were contacted by a doctor via telephone and interviewed about their child’s respiratory condition. Children with respiratory symptoms were recalled for further evaluation. Verbal consent of their participation was obtained over the phone. All children with severe HAdV pneumonia were already on regular follow-up. In addition, all parents of children who were attending clinic answered the translated Malay version of the Parent Cough-specific–Quality of Life (PC-QOL) questionnaire, as part of clinical management. The PCQOL is a validated instrument for assessing the burden of chronic cough and quality of life.[18] The questionnaire provides important information on outcome indicators and aids in evaluation of efficacy of treatment interventions. The questionnaire consists of 8 short item questions, covering quality of life domains of physical (2 items), psychological (4 items) and social (2 items) wellbeing. There are 7 options in each item, with a score scale of 1–7 per item. The higher numbers represent fewer concerns and thus, better quality of life.

### Statistical analysis

Data analysis was performed using Statistical Package for Social Science (SPSS) software version 16.0 (IBM, USA). Continuous data was expressed as mean (standard deviation [SD]) or median (interquartile range [IQR]) if not normally distributed. Chi-square test was used for comparing categorical variables between two groups and odds ratio (OR) and 95% confidence interval (CI) were reported, where appropriate. Mann-Whitney U test was used when comparing continuous (numerical) variables without normal distribution between the two groups. Logistic regression was used to determine significant factors associated with severe disease and respiratory sequelae. All tests were calculated in a two-tailed manner and significance was defined by a p value of less than 0.05.

## Results

**Fig 1:**
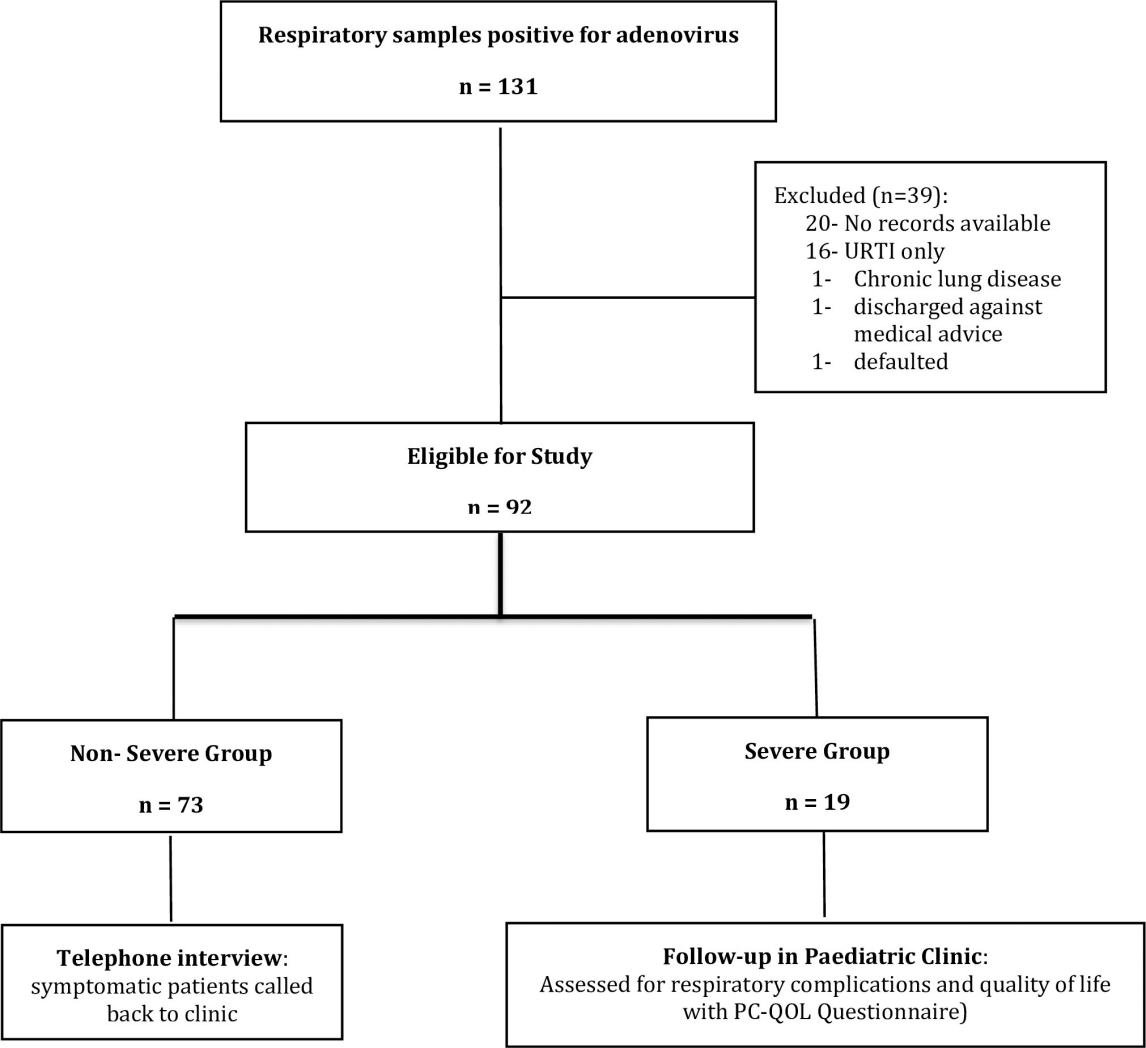
Study flow of the 92 patients with adenovirus pneumonia.

A total of 131 respiratory samples were positive for HAdV (either by IF and/or viral culture) between 1^st^ January 2011 and 31^st^ July 2013; however, 92 were included in the analysis as 39 were excluded for various reasons, as shown in the study flow, Fig 1. Nineteen children (20.6%) had severe infection while 73 (79.4%) had non-severe cases of HAdV pneumonia.

**Fig 2:**
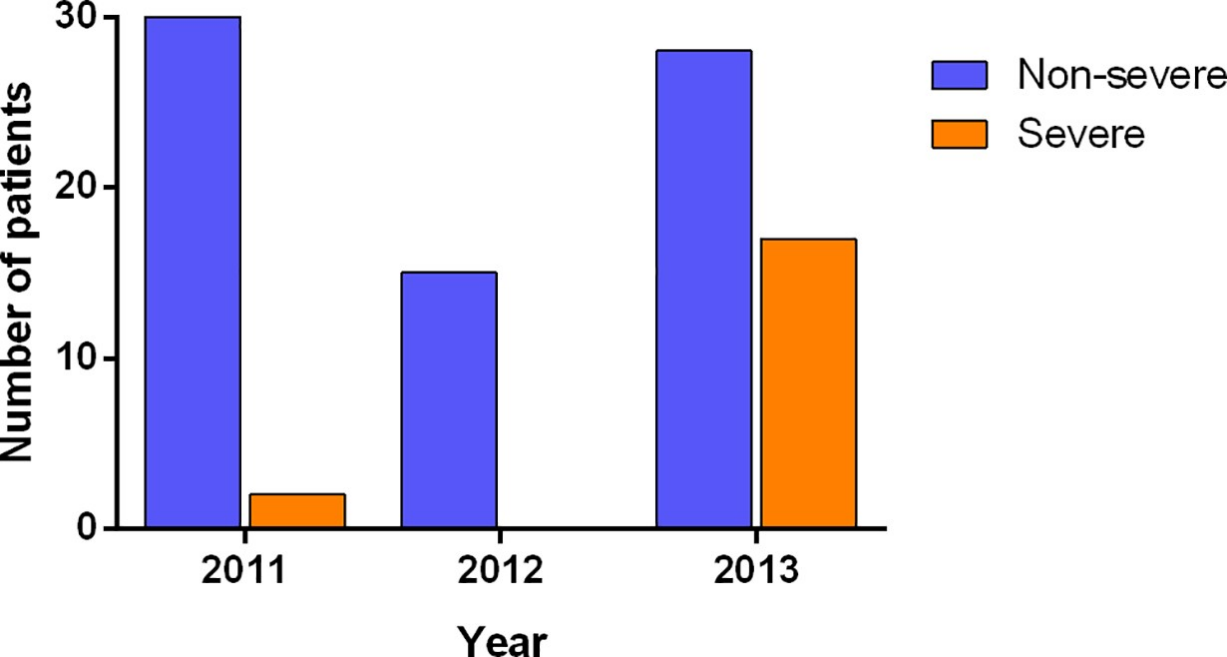
A sharp increase in the number of adenovirus infections as well as an increase in the number of severe cases was detected in 2013.

Between 2011 and 2013, there was a sharp increase in both the number and severity of HAdV cases as shown in Fig 2.

### Baseline and clinical and laboratory investigations: Severe and non-severe

Severe disease was seen in 20.6% of cases. Demographic characteristics of the patients with severe and non-severe HAdV infection are shown in Table 1.

**Table 1:** Baseline characteristics of the 92 patients with adenovirus pneumonia.

Characteristics	Total (n = 92) n (%)	Non-severe group (n = 73) n (%)	Severe group (n = 19) n (%)	P value
**Gender**				0.89
Male	52 (56.5)	41 (56.2)	11 (57.9)	
Female	40 (43.5)	32 (43.8)	8 (42.1)	
**Ethnicity**				0.089
Malay	81 (88.0)	65 (89.0)	16 (84.2)	
Chinese	3 (3.3)	1 (1.4)	2 (10.5)	
Indian	6 (6.5)	6 (8.2)	0 (0)	
Others	2 (2.2)	1 (1.4)	1 (5.3)	
**Age, years, median(IQR)**^1^	1.08(0.58–1.58)	1.08(0.52–1.64)	0.83(0.41–1.25)	z = -1.23(0.22)
< 2 years	74 (80.4)	57 (78.1)	17 (89.5)	0.218
≥ 2 years	18 (19.5)	16 (21.9)	2 (10.5)	
**Gestational age**				0.665
Term	85 (92.4)	67 (91.8)	18 (94.7)	
Preterm (< 37 weeks)	7 (7.6)	6 (8.2)	1 (5.3)	
**Birth weight**, kg, mean (S.D.) ^1^	3.03 (± 0.608)	3.04 (± 0.607)	3.00 (± 0.628)	0.825
**Previous LRTI** ^**2**^				0.053
Yes	23 (25.0)	15 (20.5)	8 (42.1)	
No	69 (75.0)	58 (79.5)	11 (57.9)	
**Co-morbid diseases** ^**3**^				0.218
Yes	8 (8.7)	5 (6.8)	3 (15.8)	
No	84 (1.3)	68 (93.2)	16 (84.2)	
**Exposure to passive smoking (n = 62)**				0.475
Yes	28 (30.4)	22 (30.1)	6 (31.6)	
No	34 (37.0)	24 (32.9)	10 (52.6)	
**Personal history ofasthma/ wheeze**				0.054
Yes	12 (13.0)	7 (9.6)	5 (26.3)	
No	80 (87.0)	66 (90.4)	14 (73.3)	
**Family history of asthma (n = 71)**				0.555
Yes	25 (27.2)	18 (24.7)	7 (36.8)	
No	46 (50.0)	36 (49.3)	10 (52.6)	
**Duration of hospitalization**, days, median (IQR)	4.5 (1.6–7.4)	4.0 (2.5–5.5)	15.0 (6.5–23.5)	**< 0.001**

^1^IQR: interquartile range; S.D.: standard deviation

^2^LRTI: lower respiratory tract infection

^3^Congenital heart disease = 3; Achondroplasia = 1; Neurological disease = 2

Most patients (80.4%) were children less than 2 years of age. There were no significant differences in demographic characteristics between those with severe and non-severe disease. There were 7 patients with underlying co-morbidities: 3 with congenital heart disease, 3 with neurological disease and 1 patient with achondroplasia. Patients in the severe group had a significantly longer duration of hospitalisation (p<0.001, z = -5.62).

Table 2 shows the clinical findings in the children with severe and non-severe HAdV infection. Children with severe disease presented with more hepatomegaly (p = 0.002, OR 5.64 [95%CI 1.89,16.84]), hepatitis (p = 0.01, OR 6.62 [95%CI 4.00,10.92]), disseminated disease (p<0.001, OR 15.41 [95%CI 4.68, 50.78]) and seizures (p = 0.03, OR 1.38 [95%CI 1.32,142.86]).

**Table 2:** Clinical characteristics of 92 children with adenovirus pneumonia.

Symptoms & Signs	Total (n = 92) n (%)	Non-severe group (n = 73) n (%)	Severe group (n = 19) n (%)	P value
**Constitutional**				
**Fever**	87 (94.6)	69 (94.5)	18 (94.7)	0.970
**Duration of fever**, median (IQR)days^1^	5.0 (3.0–7.0)	5.0 (3.0–7.0)	5.0 (1.5–8.5)	0.662
**Prolonged fever** (>7 days)	21 (22.8)	16 (21.9)	5 (26.3)	0.684
**Respiratory**				
Cough	86 (93.5)	68 (93.2)	18 (94.7)	0.803
Tachypnoea	43 (46.7)	29 (39.7)	14 (73.7)	**0.008**
Wheezing	8 (8.7)	4 (5.5)	4 (21.1)	0.054
Respiratory distress	62 (67.4)	47 (64.4)	15 (78.9)	0.122
Rhonchi	40 (43.5)	28 (38.4)	12 (63.2)	0.052
Crepitations	67 (72.8)	50 (68.5)	17 (89.5)	0.067
**Disseminated disease**				
Shock	6 (6.5)	0 (0)	6 (31.6)	**< 0.001**
ARDS^2^	7 (7.6)	0 (0)	7 (36.8)	**< 0.001**
Pulmonary haemorrhage	2 (2.2)	0 (0)	2 (10.5)	**0.041**
Hepatitis	16 (17.4)	9 (12.4)	7 (36.8)	0.773
**Extra-pulmonary**				
Vomiting ± diarrhoea	56 (60.9)	46 (63.0)	10 (52.6)	0.409
Conjunctivitis	10 (10.9)	8 (10.9)	2 (10.5)	0.294
Hepatomegaly	22 (23.9)	12 (16.4)	10 (52.6)	**0.002**
Seizures	4 (4.4)	1 (1.4)	3 (15.9)	**0.027**

^1^IQR: interquartile range

^2^ARDS: acute respiratory distress syndrome

Table 3 shows the laboratory investigation findings in children with severe and non-severe HAdV disease. Review of laboratory investigations showed that in children with severe disease, albumin (p = 0.01, z = -2.33) was significantly lower and neutrophil/lymphocyte ratio (p = 0.03, z = -2.18) was significantly higher. Viral co-infection was found in 4 patients, all in the non-severe group (RSV = 3, metapneumovirus = 1). One patient had *Haemophilus influenzae* sepsis, in the severe group. Bacterial and viral co-infection was not associated with severe adenoviral infection, as shown in Table 3.

**Table 3:** Laboratory results of 92 children with adenovirus pneumonia.

Laboratory Data	Non-severe group (n = 73) n (%)	Severe group (n = 19) n (%)	P value
**WBC**^**1**^ (× 10cells/uL) **(n = 91)**			0.160
> 15.0	28 (38.9)	4 (21.1)	
5.0–11.0	44 (61.1)	11 (57.9)	
< 5.0	0 (0)	4 (21.1)	
**Neutrophil (%), median (IQR)**^**2**^	52 (47, 61)	67 (59, 74)	**0.02** Z = -2.33
**Lymphocyte (%), median (IQR)**	34 (31, 44)	28 (20, 35)	0.05 Z = -1.98
**Monocyte (%), median (IQR)**	6.00 (5.5, 9.8)	5.04 (4.33, 7.13)	0.75 Z = -1.36
**Haemoglobin**, (g/dL) **(n = 91)**			0.076
> 11.0	48 (66.7)	9 (47.4)	
9.0–11.0	23 (31.9)	8 (42.1)	
< 9.0	1 (1.4)	2 (10.5)	
**Platelet**) **(n = 87)** < 150 × 10^3^cells/μL	1 (1.5)	3 (15.8)	**0.031**
**CRP**^**3**^ (g/L) **(n = 79), median (IQR)**	1.50 (1.32, 7.89)	2.54 (1.97, 7.32)	0.376 Z = -0.885
**Albumin** (g/l), **median (IQR)**	29 (27, 32)	24.58 (22, 27)	**0.007 Z = -2.33**
**Abnormal** (> 41 U/L)			
**ALT**^**4**^ **(n = 39)**	9 (39.1)	7 (43.8)	0.773
**Adenovirus type (n = 79)**			
**1**	13 (21.7)	1 (5.3)	0.175
**3**	9 (15.0)	1 (5.3)	0.378
**4**	3 (5.0)	0 (0)	0.369
**5**	1 (1.7)	1 (5.3)	0.300
**7**	28 (46.7)	15 (78.9)	**0.002**
**Viral co-infection**^**5**^	4 (5.5)	0 (0)	0.297
**Bacterial co-infection**^**6**^	5 (6.8)	1 (5.3)	0.803
**Radiographic findings**			
**Infiltrates ± consolidation**	69 (94.5)	17 (89.5)	0.461

^1^WBC: white blood cell

^2^IQR: interquartile range

^3^CRP: C-reactive protein

^4^ALT: alanine transaminase

^5^Viral co-infection: 3 cases of respiratory syncytial virus (RSV), 1 case of metapneumovirus

^6^Bacterial co-infection: 1 cases of *Streptococcus pneumoniae*, 3 cases of *Haemophilus influenzae*, 1 case of both *Klebsiella pneumoniae* and *Acinetobacter baumanii* in the non-severe group, 1 case of *Haemophilus influenzae* bacteraemia in the severe group.

### Human adenovirus types

**Fig 3:**
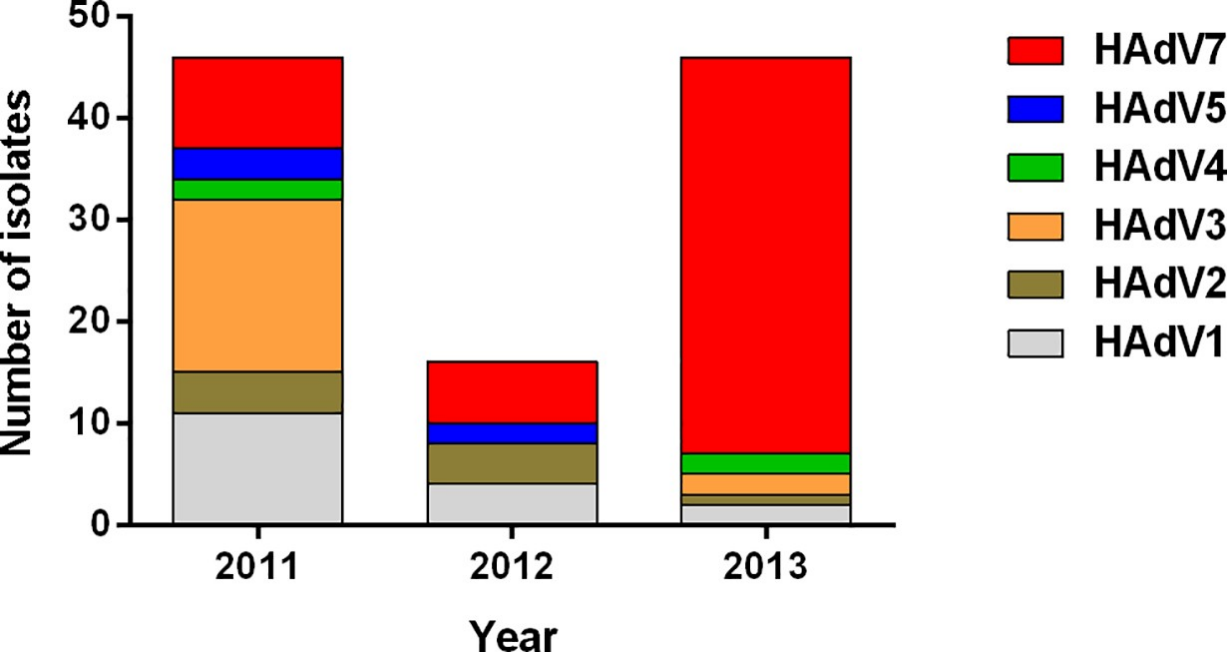
Changing human adenovirus types detected over the study period (Jan 2011 to July 2013). As HAdV3 and HAdV1 declined, HAdV7 increased to become the predominant circulating type.

Of the 131 adenovirus-positive samples, 108 were successfully sequenced and typed. Fig 3 shows the changing HAdV types over the study period from January 2011 till December 2013; in 2011, HAdV3 (37.0%) and HAdV1 (23.9%) were the commonest types, but the proportion of HAdV7 increased from 19.6% in 2011 to 37.5% in 2012 and, finally, 84.8% in 2013.

**Fig 4:**
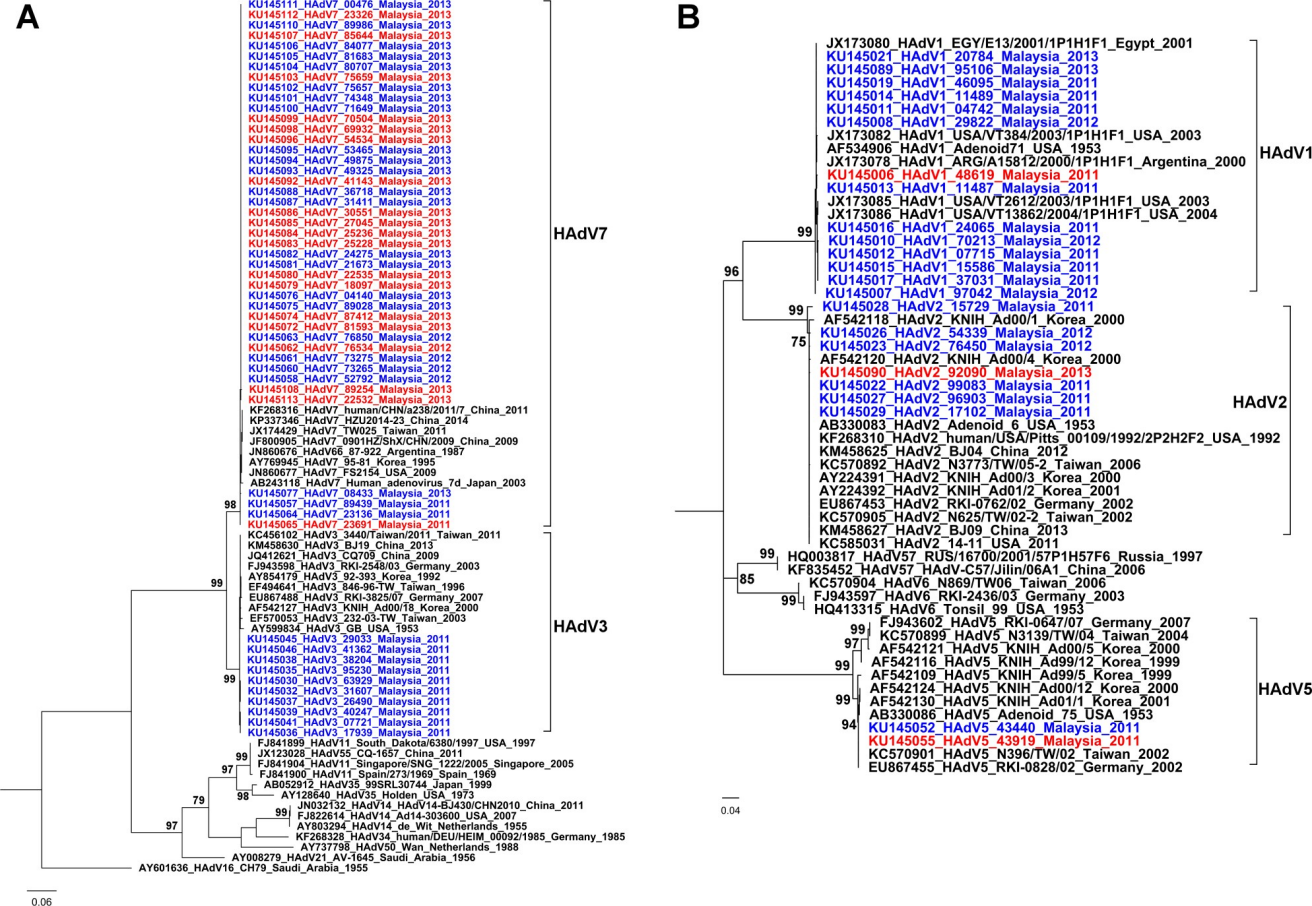
Phylogenetic analysis of partial hexon genes of human adenovirus of species B (A, 767 bases analysed) and species C (B, 800 bases). The maximum likelihood trees were constructed using the general time reversible model with proportion of invariant sites, and inferred following bootstrap analyses using 1000 replicates. Strain names are in the format: accession number_adenovirus type_strain name_country of isolation_year of isolation. The Malaysian sequences from this study are coloured red (with respiratory complications) or blue (without respiratory complications).

Of the 92 patients analysed in this study, typing was possible for 79 isolates with one patient having two isolates. Fig 4 shows the phylogenetic analyses of the HAdV isolated in this study. These comprised type 7 (n = 43, 54.4%), type 1 (n = 14, 17.7%), type 3 (n = 10, 12.7%), type 2 (n = 7, 8.9%), type 4 (n = 3, 3.8%) and type 5 (n = 2, 2.5%). The Malaysian sequences within each type were highly similar and clustered together, with no distinct intra-typic clustering associated with the presence of respiratory complications. Type 7 was the commonest identified and in univariate analysis, was significantly associated with severe infection in children (p = 0.02, OR 4.12, [95% CI 1.30,16.29]) when compared to the other types, as shown in Table 3.

### Factors associated with severe illness

In univariate analysis, duration of hospitalisation, seizures, disseminated disease (shock, ARDS, pulmonary haemorrhage), tachypnoea, hepatomegaly, neutrophil (%) count, albumin, low platelet count and HAdV type 7 (compared to the other types) were significantly associated with development of severe disease. However, in the final model, only prolonged hospitalisation was associated with severe illness (p = 0.003, OR 1.54 [95%CI 1.16, 2.06]).

### Treatment modalities

Sixty-two children (67.4%) required some form of respiratory support and 17 (18.5%) required ventilatory support (non-invasive and/or invasive). Thirteen children received intravenous IVIG (2 in the non-severe and 11 in the severe HAdV group) and 17 children received steroids (5 in the non-severe and 12 in the severe group).

### Respiratory morbidity following HAdV pneumonia

**Fig 5:**
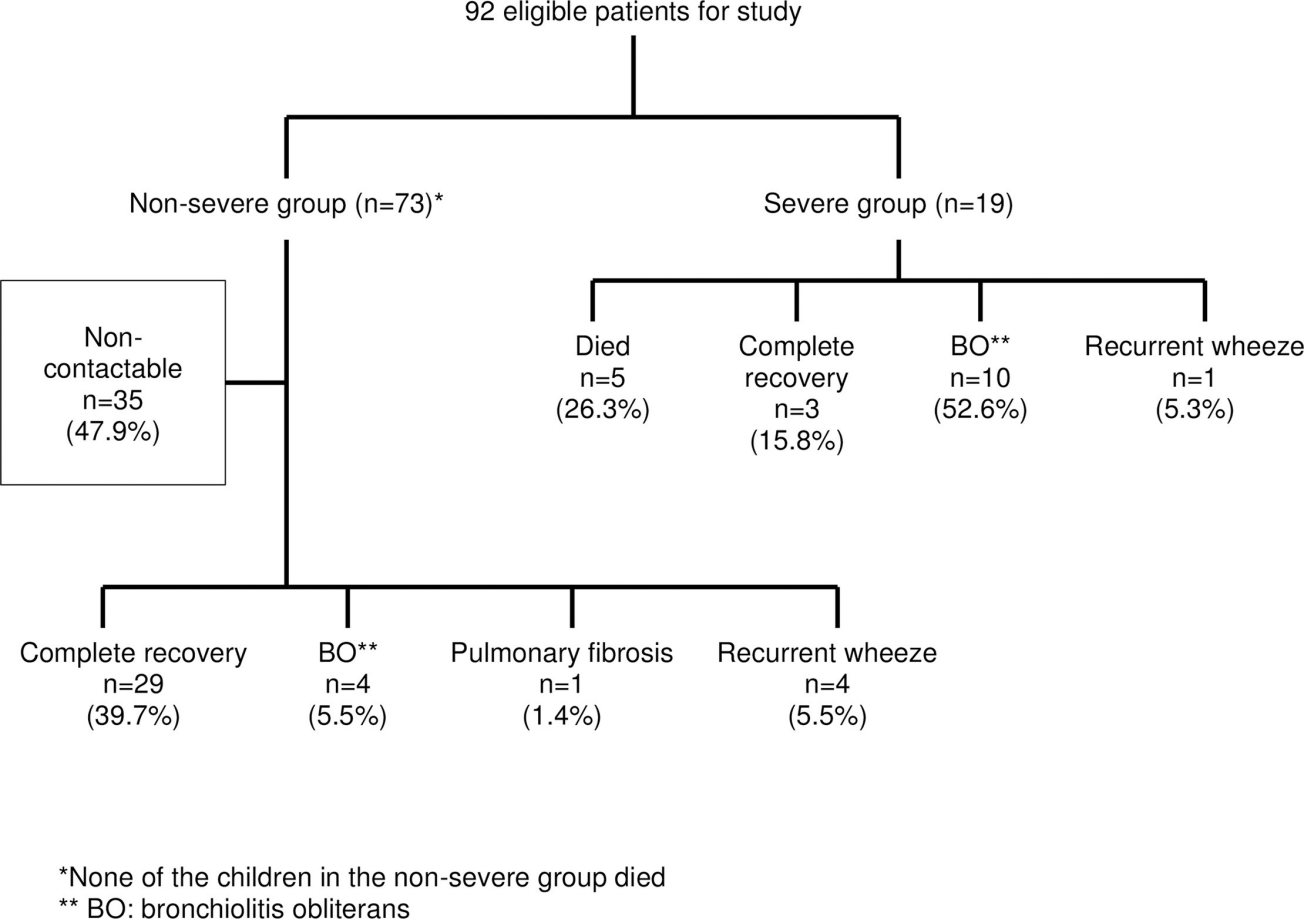
Respiratory sequelae of the 92 patients with adenovirus pneumonia.

Respiratory complications were seen in 21.7% of patients (n = 20) with an overall mortality rate of 5.4% (n = 5). Fig 5 shows the respiratory morbidity in children following HAdV pneumonia. All children who died had disseminated disease. Only three patients (15.8%) in the severe group made a complete recovery. PIBO (n = 10, 52.6%) was the commonest respiratory complication in the severe group. Five patients in the severe group needed non-invasive home respiratory support (26.5%). However, even in the non-severe group, four (5.5%) developed PIBO, one (1.4%) pulmonary fibrosis and another four (5.5%) had recurrent wheeze. Table 4 shows the demographic and clinical characteristics of the children who died (n = 5) and those with respiratory complications (n = 20).

**Table 4:** Summary of 25 patients with respiratory complications.

Index patient	Age at diagnosis	Gender	Adenovirus type	PICU^1^ admission	Respiratory support		Respiratory complications
					NIV^2^	IV^3^	
1	1 year 7 months (2013)	Male	7	No	Yes	No	BO^4^
2	3 months (2013)	Male	7	Yes	Yes	Yes	BO
3	2 years 4 months (2013)	Female	7	Yes	Yes	No	Died
4	8 months (2011)	Female	1	No	No	No	Recurrent wheeze
5	1 year (2013)	Female	7	Yes	Yes	Yes	ARDS,^5^ Pulmonary hemorrhage, BO
6	8 months 2013)	Female	7	Yes	Yes	No	BO
7	6 years 10 months (2011)	Female	4	No	No	No	Recurrent wheeze
8	8 months (2013)	Female	7	Yes	Yes	No	BO
9	7 months (2013)	Female	7	No	No	No	BO
10	1 year 1 month (2012)	Male	-	No	No	No	Recurrent wheeze
11	1 year 10 months (2011)	Female	7	No	No	No	BO
12	7 months (2013)	Female	7	No	No	No	Recurrent wheeze
13	1 year 8 months (2013)	Female	7	Yes	Yes	Yes	ARDS, pulmonary hemorrhage, died
14	1 year 6 months (2013)	Male	7	No	No	No	Respiratory failure, died
15	4 months (2013)	Male	7	Yes	Yes	Yes	BO
16	5 months (2013)	Male	7	Yes	Yes	Yes	BO
17	4 years 4 months (2013)	Male	7	No	No	No	BO
18	9 months (2013)	Male	7	No	Yes	No	BO
19	1 year 4 months (2013)	Male	7	Yes	Yes	Yes	BO
20	7 months (2011)	Female	5	Yes	Yes	Yes	Recurrent wheeze
21	1 year 4 months (2013)	Male	7	Yes	Yes	No	BO
22	1 year (2013)	Female	7	No	No	No	BO
23	10 months (2013)	Female	2	Yes	No	Yes	Respiratory failure, died
24	8 months (2013)	Female	7	Yes	No	Yes	ARDS, died
25	5 years 6 months (2013)	Male	-	No	No	No	Pulmonary fibrosis

^1^PICU: paediatric intensive care unit

^2^NIV: non-invasive ventilation

^3^IV: invasive ventilation

^4^BO: bronchiolitis obliterans

^5^ARDS: acute respiratory distress syndrome

### Respiratory morbidity 2 years following adenovirus pneumonia

We followed up with 18 of these patients, for a median (IQR) duration of 2.5 (2.3,3.1) years, in the Paediatric Respiratory clinic; 14 had severe and 4 had non-severe HAdV pneumonia. Seven (38.9%) of these patients were below the 3^rd^ centile for weight. More than half (55.6%) of these patients still reported chronic cough. Five patients (27.8%) reported significant chronic cough of three or more days in a week. Three (16.7%) patients had chronic cough severe enough to affect sleep. Almost half (44.4%) reported reduced effort tolerance. Eleven (61.1%) patients still required inhaled corticosteroids. Ten patients (55.6%) remained on oral azithromycin for its immunomodulatory benefits. Of the 13 patients (72.2%) who had hospital re-admissions for LRTIs following HAdV pneumonia, eleven (84.6%) were re-admitted three times or less while two patients (15.4%) reported hospital re-admission on more than three separate occasions.

Eleven (61.1%) of these patients still required inhaled corticosteroids. Ten patients (55.6%) remained on oral azithromycin for its immunomodulatory benefits. The total PC-QOL mean score was 3.93 ± 0.36. One third (n = 7) had low PC-QOL scores (PC-QOL score < 4) indicating reduction in quality of life. Mean scores for each domain ranged between 3.17 (SD 2.13) - 4.48 (SD 2.37) indicating that parents were “some of the time”—“quite often” disturbed by their children’s cough.

While use of methylprednisolone and IVIG did not change the respiratory outcome, 4 out of the 5 patients who died did not receive either treatment.

### Risk factors associated with development of respiratory complications

Table 5 shows all the factors investigated in determining significant associations with respiratory morbidity. However, after multivariate analysis, the final model showed that family history of asthma (p = 0.006, OR 14.96 [95% CI 2.15–104.05]), need for either invasive or non-invasive ventilatory support (p< 0.001, OR 153.77 [95% CI 10.07–2.32E]) and HAdV type 7 (compared to the other types) (p = 0.025, OR 9.00, [95% CI 1.34–60.34]) were independent factors associated with the development of respiratory complications. Table 6 shows the multivariate analysis of risk factors associated with respiratory complications post-adenovirus pneumonia

**Table 5:** Baseline characteristics, symptoms and signs of the 92 patients with adenovirus pneumonia with and without respiratory complications.

Characteristics	Total (n = 92) n (%)	Patient with respiratory complications (n = 25) n (%)	Patient without respiratory complications (n = 67) n (%)	P value (Z score)	Odds ratio 95% (Confidence interval)
**Gender**				0.139	0.50 (0.20,1.26)
Male	52 (56.5)	11 (44.0)	41 (61.2)		
Female	40 (43.5)	14 (56.0)	26 (38.8)		
**Ethnicity**				0.821	NA
Malay	81 (88.0)	22 (81.0)	59 (88.1)		
Chinese	3 (3.3)	1 (4.0)	2 (3.0)		
Indian	6 (6.5)	1 (4.0)	5 (7.5)		
Others	2 (2.2)	1 (4.0)	1 (1.5)		
**Age, years, median (IQR)**^**1**^	1.08 (0.58–1.58)	1.00 (0.62–1.63)	1.08 (0.67–1.67)	0.627 (-0.44)	NA
< 2 Years	74(80.4)	21(84.0)	53(79.1)	0.770*	1.39 (0.49,4.70)
≥ 2 Years	18 (19.5)	4 (16.0)	14(20.9)		
**Gestational age** Preterm (< 37 weeks)	7 (7.6)	0 (0.0)	7 (10.4))	0.185*	0.71 (0.62, 0.81)
Term	85 (92.4)	25 (100.0)	60 (89.6)		
Preterm	7(7.6)	0(0.0)	7(10.9)		
**Previous chest** **infection**				**0.042**	2.77 (1.02,7.55)
Yes	23 (25.0)	10 (40.0)	13 (19.4)		
No	69 (75.0)	15(60.0)	54 (80.6)		
**Passive smoking** **(n = 62)**					
Yes	28(30.4)	13(52.0)	15(22.4)	**0.182**	2.06 (0.72,6.05)
No	34 (37.0)	10 (40.0)	24 (35.8)		
**Personal history of** **asthma/wheeze**				0.810*	3.21 (0.93,11.13)
Yes	12 (13.0)	6 (24.0)	6 (9.0)	0.810*	
No	80 (87.0)	19 (76.0)	61 (91.0)		
**Family history of** **asthma (n = 71)**				**0.005**	4.47 (1.56,13.39)
Yes	25 (27.2)	14 (56.0)	11 (16.4)		
No	46 (50.0)	10 (44.0)	36 (53.7)		
**Duration of fever**, median, (IQR)days	5.0 (3.0–7.0)	5.0 (3.0,7.0)	5.0 (3.0,7.0)	0.443 (-0.70) 0.797*	NA
**Prolonged fever** (>7 days)	21 (22.8)	5 (20.0)	16 (23.9)		0.80 (0.26,2.47)
**Respiratory signs**					
Respiratory distress	43 (46.7)	21 (84.0)	42 (62.7)	0.050	3.13 (0.91,10.15)
Rhonchi	16 (43.5)	16 (64.0)	24 (38.8)	0.015	3.19 (1.22,8.30)
Crepitations	21 (72.8)	21(84.0)	41 (68.7)	0.141	2.40 (0.73,7.87)
**Extra-pulmonary**					
Vomiting ± diarrhoea	56(60.9)	15(60.0)	41(61.2)	0.417	0.95 (0.37,2.43)
Hepatomegaly	22(23.9)	10(40.0)	12(17.9)	0.027	3.06 (1.11,8.43)
Seizures	4(4.4)	3(12.0)	1(1.5)	0.030	9.00 (0.89,91.04)
Disseminated disease	22(23.9)	13(52.0)	9(13.4)	<0.001	6.98 (2.44, 20.01)
Hepatitis	16 (17.4)	3 (12.0)	0 (0.1)	0.018*	**4.05** **(2.82,5.81)**
**Investigations**					
Neutrophil/ lymphocyte ratio	1.581 (0.84,2.88)	2.48 (1.46,4.76)	1.23(0.76,2.19)	**0.001 (-3.42**)	NA
Platelet	278 (213,356)	292 (179,356)	278(227,356)	0.506 (-0.67)	NA
C-reactive protein	1.75 (0.98,4.98)	2.14 (0.88,4.57)	1.65(1.00,5.12)	0.920 (-0.13)	NA
Albumin	28 (25,32)	26 (23,29)	31 (29,33)	**0.015 (-2.43)**	NA
ALT^2^	39 (24,84)	40 (25,82)	38 (23,113)	0.990 (-0.13)	NA
**Adenovirus type(n = 79)**				**0.002**	5.94 (1.78,19.80)
Type 7	43 (54.4)	19 (82.6)	24 (44.4)		
Non-7 types	36 (45.6)	36 (45.6)	30 (55.6)		
**Treatment**					
PICU^3^ admission	14 (15.2)	13 (52.0)	1 (1.5)	**<0.001**	71.5 (8.54,598.62)
Ventilation^4^	47 (51.2)	21 (84.0)	26 (38.9)	**<0.001**	8.28 (2.55, 26.86)
Steroids	13 (14.1)	12 (48.0)	1 (1.5)	**<0.001**	11.49 (3.44, 38.10)
**Duration of hospitalization**, days, median (IQR)	4.5 (1.6–07.40)	10.0 (4.0–22.50)	4.0 (3.0–6.0)	**< 0.001** **(-3.83)**	**NA**

*Fishers exact test

^1^IQR: interquartile range

^2^ALT: alanine transferase

^3^PICU: Paediatric Intensive Care Unit

^4^invasive or non-invasive ventilation

**Table 6:** Multivariate analysis of risk factors associated with respiratory complications post-adenovirus pneumonia.

Risk factors	Patients with respiratory complications n = 25 (%)	Patients without respiratory complications n = 67 (%)	Multivariate analysis	
			Adjusted OR (95% CI)	P value
Family history of asthma	14 (56.0)	11 (16.4)	14.96 (2.15–104.05)	**0.006**
Need for invasive or non-invasive ventilation	15(60.0)	2(3.0)	153.77 (10.07–2.35E)	**<0.001**
Adenovirus type versus other types	24(96.0)	19(28.0)	9.00 (1.34–60.34)	**0.024**

## Discussion

This comprehensive report on HAdV pneumonia in Malaysian children summarises their clinical presentation, identified HAdV types, treatment, risk factors for severe disease and both short- and medium-term respiratory complications. We found that one in five children admitted with HAdV pneumonia had severe disease and 22% developed respiratory complications of which PIBO was the commonest problem. Type 7 was the commonest type detected. Family history of asthma, need for ventilation(invasive and non-invasive) and type 7 were independent factors associated with respiratory complications.[15]

Epidemics of HAdV pneumonia have been reported since 2011, in both children and adults.[6,8,9,19–21] While predominant types circulating at a given time differ among countries or regions, and change over time [22], type 7 was the main type reported in China, Taiwan and Singapore between 2011 and 2013. Among 632 HAdV cases reported during the Taiwan outbreak in 2011, HAdV3 was predominantly seen in children with upper respiratory tract infections while HAdV7 was seen in cases that required PICU care or died.[23] As seen in our study, neighbouring Singapore also saw the emergence of HAdV type 7 as the predominant type between 2011 and 2013. [21] An earlier study from our centre found that of the HAdV isolates from 1999 to 2005, 70% of isolates were human adenovirus C (HAdV-1, HAdV-2, HAdV-5 and HAdV-6) [24], showing a longer term shift to species B (HAdV3 and HAdV7) in 2011–2013. [24]

In this study, the majority of children were under 2 years old. This is interesting as recent studies from Taiwan and China found that rate of HAdV infection increased with age.[23,25] However, in the study from Taiwan, only 12% of patients had LRTIs. The experiences in Singapore, where most paediatric patients infected with HAdV were < 2 years old. [21] and in Seremban, Malaysia, where in 2015 the median age of inpatients with HAdV was14 months, are more similar to ours.[4] It has been reported that young children are at increased risk of severe HAdV infections. [26]

Treatment and outcome were the main concerns in this study. Twenty percent of children with HAdV pneumonia had a severe infection, which we defined as requiring NIV or IV or PICU admission or death. The case fatality was 5.4%. Our severity and case fatality are much higher than that previously mentioned in the study from Seremban, Malaysia, which were 11% and 2.6% respectively. [5] This could be explained as we are a tertiary referral centre that accepts ill patients from peripheral hospitals. While there were many factors associated with severe infection in univariate analysis, only hospital duration was an independent association. Rajkumar et al identified age < 2 years old and presence of significant comorbidities as independent risk factors for severe disease. We did not find this association with age and in this study, we excluded those with serious comorbidities. In the outbreak in Taiwan, authors found an association between severe disease and presence of pleural effusion. Pleural effusion was seen in only 2 children in this study (one in a severe HAdV and one in a non-severe HAdV) and none required tube thoracostomy drainage. Immunocompromised children and children with comorbidities have also been reported to be at increased risk of severe HAdV infections[6,19,27]. Many laboratory features have been associated with severe HAdV infection e.g. leucopenia,[23] thrombocytopenia[23] and a positive blood culture.[20]

In this study, 22% of children developed respiratory complications. Even in the non-severe group, 12.3% (9 out of 73) children developed respiratory complications. This is much higher than what has been previously reported locally, where only one child developed PIBO.[5] However in other reports, especially from Latin America, respiratory complications of PIBO range between 36 to 47% and mortality rates can be as high as 15%[1,12] The need for NIV, PICU admission and family history of asthma were independent risk factors for respiratory complications. Most of our patients who had respiratory distress or impending respiratory failure would have received NIV. Therefore, children with severe respiratory compromise from HAdV have an increased risk of respiratory complications. As for family history of asthma, a severe infection could trigger asthma, especially if there is a genetic predisposition. Castro-Rodriquez from Chile, who followed up 45 children with HAdV pneumonia for 5 years also found that significant respiratory compromise (intensive care admission, need for mechanical ventilation and for oxygen therapy, and systemic corticosteroid and beta agonist use) was associated with risk for PIBO.[1]Hence his results concur with ours. In China, hypoxaemia was the only factor associated with risk for PIBO.[28]

No antiviral treatments are currently licensed for treatment of severe HAdV disease.

In this study, we used IVIG and IV MTP, but not in a randomised fashion. While we did not see any effect on subsequent respiratory complications, 4 out of the 5 who died from HAdV did not receive these medications. Takahashi et al. reported that use of pulse methylprednisolone (25mg/kg/day) for 3 days, in a case of severe HAdV pneumonia (type 3) with hypercytokinemia i.e raised lactate dehydrogenase, ferritin, interferon-gamma and interleukin-6, resulted in relief of respiratory distress. [29] He also suggested that serum Krebs von den Lungen-6 (Kl-6) could be used as a marker of future lung disease. Cidofivir is occasionally used in immunocompromised children with severe HAdV. It has been shown to clear the virus from blood, however mortality despite its use stands at 10–70% and it is nephrotoxic.[30] There have also been case reports of use of ribavirin in immunocompromised children with HAdV, [31] as well as successful use of oral ribavirin in an adult with respiratory compromise.[32]However use of only antivirals may not suffice, and other adjuvant medication like MTP and IVIG may be necessary.

This is the first study looking at the quality of life in children with post-HAdV lung disease. We found that more than half of the children under follow-up had chronic cough, more than a third were underweight, nearly half still had reduced effort tolerance and as many as 72% required unscheduled healthcare visits. Most parents were still concerned about their child’s cough. More work is needed to address the long-term consequences of this viral infection, which may lead to reduced lung function that is not fully reversible.[1]

Limitations of our study are recognised including the small number of patients, not using polymerase chain reaction to detect HAdV which would increase the sensitivity of virus detection, the inability to contact all patients who had HAdV pneumonia and the inability to type all the 92 detected HAdV virus. However, the strength of this study is that it has comprehensive data including clinical and laboratory parameters, HAdV type, respiratory outcomes, and a follow-up of up to 2 years, for a fairly large number of children.

## Conclusion

In conclusion, during the sharp increase of HAdV infection in Malaysia between 2011 and 2013, the majority of children admitted for HAdV pneumonia were less than 2 years old. One in five children had severe disease and the case fatality rate was 5.4%. HAdV 7 was the most frequently detected type isolated amongst children with severe pneumonia and those with persistent respiratory sequelae. Severe disease was associated with prolonged hospitalisation. Twenty-two percent developed respiratory complications, commonest being bronchiolitis obliterans (15.2%) and recurrent wheeze (5.4%). Presence of severe respiratory compromise, isolation of HAdV type 7 and family history of asthma, were independent risk factors associated with respiratory sequelae. Children with respiratory complications reported significant reduction in quality of life. There is a lack of good and adequately powered studies to determine the best treatment for this disease which has a high mortality and significant morbidity.
