# Correction: Epidemiology, clinical presentation and respiratory sequelae of adenovirus pneumonia in children in Kuala Lumpur, Malaysia

**DOI:** 10.1371/journal.pone.0209720

**Published:** 2018-12-19

**Authors:** Limin Li, Yen Yen Woo, Jessie Anne de Bruyne, Anna Marie Nathan, Sze Ying Kee, Yoke Fun Chan, Chun Wei Chiam, Kah Peng Eg, Surendran Thavagnanam, I-Ching Sam

The first author's name is spelled incorrectly. The correct name is: Limin Li. The correct citation is: Li L, Woo YY, de Bruyne JA, Nathan AM, Kee SY, Chan YF, et al. (2018) Epidemiology, clinical presentation and respiratory sequelae of adenovirus pneumonia in children in Kuala Lumpur, Malaysia. PLoS ONE 13(10): e0205795. https://doi.org/10.1371/journal.pone.0205795
